# Loss of Adipocyte STAT5 Confers Increased Depot-Specific Adiposity in Male and Female Mice That Is Not Associated With Altered Adipose Tissue Lipolysis

**DOI:** 10.3389/fendo.2022.812802

**Published:** 2022-04-07

**Authors:** Allison J. Richard, Hardy Hang, Timothy D. Allerton, Peng Zhao, Tamra Mendoza, Sujoy Ghosh, Carrie M. Elks, Jacqueline M. Stephens

**Affiliations:** ^1^ Adipocyte Biology Laboratory, Pennington Biomedical Research Center, Baton Rouge, LA, United States; ^2^ Cardiovascular and Metabolic Disease Program and Center for Computational Biology, Duke-NUS Graduate Medical School, Singapore, Singapore; ^3^ Department of Biological Sciences, Louisiana State University, Baton Rouge, LA, United States

**Keywords:** adipose, STATs, sexual dimorphism, growth hormone, transcription

## Abstract

STATs (Signal Transducers and Activators of Transcription) 5A and 5B are induced during adipocyte differentiation and are primarily activated by growth hormone (GH) and prolactin in fat cells. Previous studies in mice lacking adipocyte GH receptor or STAT5 support their roles in lipolysis-mediated reduction of adipose tissue mass. Male and female mice harboring adipocyte-specific deletion of both STAT5 genes (STAT5^AKO^) exhibit increased subcutaneous or inguinal adipose tissue mass, but no changes in visceral or gonadal fat mass. Both depots display substantial increases in adipocyte size with no changes in lipolysis in adipose tissue explants. RNA sequencing analysis of subcutaneous adipose tissue and indirect calorimetry experiments reveal sex-dependent differences in adipose gene expression and whole-body energy expenditure, respectively, resulting from the loss of adipocyte STAT5.

## Introduction

Adipocytes, the defining cells within adipose tissue, regulate whole-body energy homeostasis. In the obese state, which is characterized by excessive adipose tissue accumulation, the inability to safely store lipids in adipose tissue contributes to elevated lipid levels in the circulation (dyslipidemia) and/or liver (hepatic steatosis). Obesity has become a global epidemic and is a major risk factor for type diabetes mellitus (T2DM) and other chronic diseases. However, some people with obesity are metabolically healthy (1), and subcutaneous fat can protect against metabolic dysfunction (2). Mouse models and humans with reduced growth hormone (GH) signaling exhibit increased subcutaneous adiposity but improved metabolic health (3–7).

The Janus kinase/signal transducer and activator of transcription (JAK/STAT) pathway mediates cellular signaling of many hormones and cytokines (8). In mice, STAT5A and 5B proteins have 96% similarity despite being encoded by separate genes (9); they each have some essential and redundant signaling roles (10, 11). Although STAT5 proteins are activated by numerous cytokines/hormones, reported phenotypes of STAT5-null mice support the requirement for STAT5 only in proper GH and prolactin functions (10).

STAT5 polymorphisms associate with altered cholesterol metabolism (12), body weight (9), and lipid metabolism (13). In addition, STAT5 proteins regulate adipocyte development [reviewed in (14)], fat mass (10), and lipid metabolism (13), through mechanisms that remain enigmatic. JAK2-STAT5 signaling in various tissues contributes to obesity and T2DM (15), and disruption of the adipocyte JAK2-STAT5 pathway (16) improves systemic metabolism and liver function (17–19).

To understand the metabolic functions of adipocyte STAT5, we generated mice lacking both *Stat5* genes in adipocytes (STAT5^AKO^). Compared to littermate controls, STAT5^AKO^ mice fed chow diet had pronounced increased subcutaneous fat mass, similar to mammalian models with deficient GH signaling (3–7), and female STAT5^AKO^ mice had decreased insulin levels and improved insulin sensitivity as indicated by homeostasis model assessment of insulin resistance (HOMA-IR). Although both sexes of STAT5^AKO^ mice had increased adiposity, assessment of energy expenditure and whole adipose tissue gene expression revealed that loss of adipocyte STAT5 confers sexually dimorphic responses in mice.

## Materials and Methods

### Animals

Mice with *Stat5a* and *Stat5b* genes flanked by loxP sites (floxed) (20) were bred to adiponectin-Cre (AdipoQ-Cre) mice to produce offspring heterozygous for floxed STAT5 alleles and hemizygous for AdipoQ-Cre (STAT5^fl/+:AdipoQ-Cre/+^), which were then crossed with STAT5^fl/fl:+/+^ mice to create STAT5^AKO^ mice (STAT5^fl/fl:AdipoQ-Cre/+^) and control littermates (STAT5^fl/fl:+/+^). All mice were on a C57BL/6J background. Unless otherwise stated, mice were housed in a temperature (22 ± 2°C)- and humidity-controlled (45–55%) room under a 12-h light/dark cycle with free access to food and water. Mice used for thermoneutrality experiments were housed at 28°C. All mice were 6 weeks to 11 months of age and fed standard chow (13% kcal from fat), breeder chow (26% kcal from fat), low-fat diet, no sucrose (LFD; 10% kcal from fat), or high-fat diet (HFD; 60% kcal from fat), as specified in the figure legends. Mice were humanely euthanized by carbon dioxide inhalation followed by cervical dislocation. All regulations of the Institutional Animal Care and Use Committee at Pennington Biomedical Research Center were strictly followed, and experiments were performed under approved protocols 977 and 985.

### Body Composition Measurements (NMR)

Body composition was measured by NMR, and adiposity was calculated as total fat mass divided by total BW x 100.

### Blood Glucose and Serum Analyses

Whole-blood and serum samples were collected following a 4-hour fast. Whole-blood was collected *via* a tail prick, and glucose levels were measured using a Breeze 2 glucometer. For serum analyses blood was collected *via* cardiac puncture at the time of euthanasia. Serum GH, insulin-like growth factor-1 (IGF-1), and insulin were measured by ELISA according to manufacturer instructions. Serum glycerol, non-esterified fatty acid (NEFA), triglyceride (TG) levels were measured using colorimetric assays and a microplate reader. For TG measurements, blood was collected from overnight-fasted mice using capillary blood collection tubes. HOMA-IR was calculated from glucose and insulin concentrations as follows: fasting glucose (mg/dl) × fasting insulin (µU/ml)/405 (21, 22).

### RNA Isolation and RT-qPCR Quantification

Tissues were flash frozen in liquid nitrogen and stored at –80°C. RNA was extracted by homogenization in TRIzol reagent, followed by chloroform extraction according to the TRIzol reagent manufacturer’s protocol and further column purification using the RNeasy mini kit. RNA was quantified using a NanoDrop spectrophotometer. cDNAs were synthesized and expression levels determined as previously described (23). Primer sequences are listed in **Table S1**.

### Immunoblotting

Frozen tissues were homogenized in immunoprecipitation (IP) buffer containing protease and phosphatase inhibitors as previously described (24) with the addition of 100µM sodium fluoride, 2X protease, and 4X phosphatase inhibitors. Lysates were clarified and protein concentrations were determined by bicinchoninic acid (BCA) protein assay. Protein extracts (50 µg total protein per lane) were separated on 7.5 or 10% sodium dodecyl sulfate (SDS)-polyacrylamide gels, transferred to nitrocellulose membranes, probed with primary antibodies (**Table S2**), and visualized as previously described (24, 25).

### Adipose Tissue Fractionation

Adipose fractionation was performed using a protocol adapted from (26). Briefly, excised adipose tissue was weighed, immediately placed on ice in DMEM + 5% heat-inactivated fetal bovine serum (FBS), minced, then incubated with Type 1 collagenase at 37°C in a shaking water bath until fully digested. Following centrifugation, the floating adipocyte layer was recovered and diluted 1:4 in 5X IP buffer; the pellet was resuspended in ACK lysing buffer for red blood cell lysis, filtered by successive passages through cell strainers (pore sizes of 100µm and 40µm), washed with PBS, and centrifuged at 10,000 x g to yield the stromal vascular fraction (SVF) pellet, which was subsequently resuspended in 1X IP buffer. Following a single freeze/thaw cycle at -80°C, the resuspended adipocyte fraction and SVF were each passed through a 20-gauge needle 10 times and then centrifuged at 10,000 x g for 10 min. The aqueous layer between the floating lipid and pellet of the adipocyte fraction, and the supernatant of the SVF were used for immunoblotting analyses. All centrifugations were performed at 4°C.

### Adipose Histology and Adipocyte Size Analysis

Excised adipose tissue was fixed in 10% neutral buffered formalin for a minimum of 24 hours. Tissues were processed, embedded, stained, and imaged, and adipocyte area was calculated from > 3000 cells per mouse per depot as previously described (27).

### *Ex Vivo* Lipolysis

*Ex vivo* lipolysis assays were performed as described in (28). Mice were fasted for 4 hours prior to euthanasia and white adipose tissue (WAT) excision. Briefly, gonadal WAT (gWAT) explants (10 – 20 mg, in triplicate) were incubated in 200 µl DMEM containing 2% fatty acid (FA)-free BSA, with or without 10 µM isoproterenol in 96 well plates at 37°C in a 5% CO_2_ and 95% humidified incubator for two hours. Released glycerol and NEFA were quantified from the conditioned medium and normalized to tissue weight.

### Indirect Calorimetry/Energy Expenditure Analyses

Mice were weaned onto standard chow diet at 3 WOA. Beginning at 7 WOA, mice were either switched to a characterized low-fat diet or remained on chow diet for the duration of indirect calorimetry assessment in the metabolic cage system starting at 10-11 WOA. Oxygen consumption and carbon dioxide production were continuously monitored for 3 days to calculate estimate of energy expenditure. Cages were maintained with 12-hr light/dark cycles. Locomotor activity (pedometers) was determined by calculating the sum of all detectable motion determined using an infrared photocell beam interruption technique (> 1 cm/s along X-, Y-, or Z-axis) over the continuous monitoring period.

### RNA-Sequencing and Analysis

For RNA sequencing analysis, RNA was isolated from subcutaneous iWAT of 6-week-old mice as described for RT-qPCR quantification. RNA integrity numbers >7 were confirmed using a Bioanalyzer RNA 6000 chip (Agilent). Sequencing libraries were constructed using a Quant-Seq 3’ mRNA-Seq Library Prep kit. Each sample was prepared with a unique sample index. Completed libraries were analyzed on the Bioanalyzer High Sensitivity DNA chip (Agilent) to verify correct library size. All libraries were pooled in equimolar amounts and sequenced on the NextSeq 500 sequencer at 75bp forward read and 6bp forward index read. Primary data analysis was performed using the Lexogen QuantSeq pipeline V1.8.8 on the Bluebee platform for quality control, mapping, and read count tables. Raw and processed data are deposited in the GEO database (accession number GEO: GSE113939).

Raw count matrices of RNA sequencing data were obtained *via the Rsubread* (29) package in R, and further processed for gene quantification and identification of differentially expressed genes using the *limma* package (30). Sequencing data were aligned to the GRCm38.84 mouse genome (mm10). Genes with at least one count per million (CPM) reads in 6 or more samples were retained for further analysis, resulting in 15118 genes. Gene counts were log2 transformed and normalized for sequencing depth *via* the TMM method (31). The mean-variance relationship of gene-wise standard deviation to average logCPM gene signal was assessed *via* the ‘voom’ method (32) to generate precision weights for each individual observation. The logCPM values and associated precision weights were subsequently utilized to generate empirical Bayes moderated t-statistics estimates for identification of differentially expressed genes. To control for multiple testing, false discovery rates were computed and genes with an adjusted p-value ≤ 0.05 were deemed differentially expressed. Venn analysis of gene lists were conducted *via* Venny (https://bioinfogp.cnb.csic.es/tools/venny/index.html).

Pathway enrichment analysis on differentially expressed genes was conducted *via* gene set enrichment analysis or GSEA (33, 34), using a custom pathway database consisting of KEGG pathways (obtained from MSigDB) plus some user-defined custom gene sets. Due to the genomic scale of the study, we used all genes annotated in the genome as the background set for the GSEA (as recommended in the DAVID tutorial (https://david.ncifcrf.gov/helps/FAQs.html#16).

### Quantification and Statistical Analysis

All data are expressed as mean ± SEM. GraphPad Prism 8.0 and JMP version 14 were used for statistical analyses. For comparisons between two independent groups, a Student’s t test was used and p < 0.05 was considered statistically significant. For comparisons between three or more groups, two-way ANOVA with Tukey’s multiple comparisons testing was performed. Analysis of covariance (ANCOVA) was used to determine differences between groups in energy expenditure using adjustments for fat and fat-free mass. All sample sizes, statistical test methods, and p-values are listed in the figure legends.

### Data and Resource Availability

STAT5^AKO^ mice generated in this study are available from the corresponding author with a completed Material Transfer Agreement, if mice are still being bred at the time. If the STAT5^AKO^ mice are unavailable at the time of the request, they can be easily re-created by breeding STAT5^fl/fl^ mice, which we can provide with permission from Dr. Lothar Hennighausen, with Adipoq-Cre mice (B6.FVB-Tg(Adiopq-cre)1Evdr/J), which are available from Jackson Laboratory (Stock No. 028020). We are glad to share requested mice with reasonable compensation for shipping. Further information and requests for resources and reagents should be directed to and will be fulfilled by the corresponding author. Commercially available resources utilized in these studies are described in **Table S2**. RNA-Seq data are available at in the GEO repository under the accession number GEO: GSE113939.

## Results

### Confirmation of Adipocyte-Specific STAT5A and 5B Knockout Status in STAT5^AKO^ Mice

**Figure 1:**
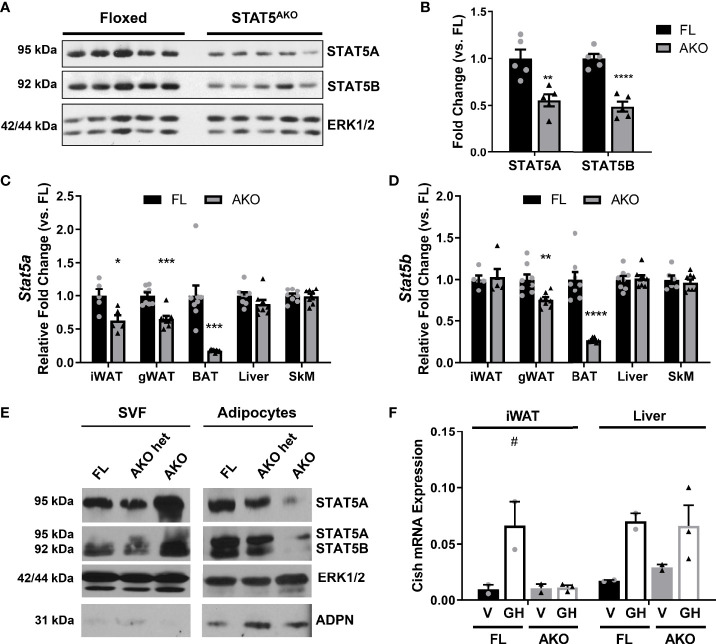
Expression of STAT5A and 5B is knocked down in adipose tissue and adipocytes of STAT5^AKO^ female mice. Female STAT5^AKO^ (AKO) mice and their floxed (FL) littermate controls were euthanized at 3 **(A–D)**, 4 **(E)**, or 11 **(F)** months of age, and tissues were immediately collected for protein or gene expression analyses **(A–D, F)** or cellular fractionation **(E)**. **(A)** Immunoblot of proteins resolved from iWAT samples for 5 mice of each genotype. **(B)** Quantification of band intensities from A (n = 5 per group). Band intensities were normalized to the loading control ERK1/2 and represented as fold change relative to FL mice. **(C, D)** *Stat5a* and *Stat5b* gene expression measured by RT-qPCR for the indicated tissues (n = 5 – 8 mice per group). **(E)** Gonadal white adipose tissue was removed from female STAT5^AKO^, heterozygous STAT5^AKO^ (het AKO), and homozygous floxed control (FL) mice, fractionated into adipocytes and stromal vascular fraction (SVF), and 100 µg (SVF) or 200 µg (adipocyte) protein subjected to western blotting. Also shown are ERK1/2 as a loading control and adiponectin (ADPN) as an adipocyte marker. For each protein, the SVF and adipocyte samples were run on the same gel and the images were from the same exposure of the same blot. **(F)** Eleven-month-old female mice were injected with 1.5mg/kg mGH or vehicle (V; 0.1% BSA/PBS) for 30 minutes prior to euthanasia and tissue collection. *Cish* gene expression measured by RT-qPCR is shown (n = 2 – 3 mice per group). Significance was determined by *t*-test for FL versus AKO comparisons in A-D) and is denoted as *p < 0.05, **p < 0.01, ***p < 0.001, ****p < 0.0001. For F, a 2-way ANOVA was used to assess treatment/genotype and tissue variables with Tukey’s *post-hoc* multiple comparisons test to compare all treatment/genotype groups for each tissue; ^#^ denotes p < 0.05 for GH versus V comparisons. See also **Figure S1**.

STAT5A and 5B proteins (**Figures 1A, B**) and mRNA (**Figures 1C, D**) were reduced, but not absent, in whole fat from STAT5^AKO^ mice. We examined gene expression in several fat pads as well as liver and skeletal muscle (**Figures 1C, D**). *Stat5a* mRNA was 50% lower in iWAT and gWAT of knockout mice, and *Stats 5a* and *5b* mRNA in brown adipose tissue (BAT) were both reduced by >60%. There were no changes in *Stat5* gene expression in liver or skeletal muscle of female mice. Although *Stat5b* gene expression was not reduced in iWAT (**Figure 1D**), STAT5B protein levels were significantly reduced (**Figures 1A, B**), and we observed decreased *Stat5b* mRNA levels in iWAT from subsequent cohorts of STAT5^AKO^ mice (data not shown).

Since STAT5 is also present in the stromal vascular fraction (SVF) of adipose tissue (AT), we fractionated fat and observed the expected decreases in STAT5 proteins in the adipocyte fractions from AKO heterozygotes (het) and STAT5^AKO^ mice (**Figure 1E**). We also consistently observed elevated STAT5A protein levels in the SVF of STAT5^AKO^ mice. There were no changes in STAT5 protein levels in liver or skeletal muscle in STAT5^AKO^ female mice (data not shown). Growth hormone (GH) rapidly induces *Cish* gene expression in a STAT5-dependent manner (35). Acute GH injection increased *Cish* mRNA expression in the livers of both floxed and STAT5^AKO^ mice, but only in iWAT from floxed mice (**Figure 1F**). We made similar observations in male mice (**Figure S1**). Collectively, our results demonstrate Cre-mediated excision of *Stat5a/b* only in mature adipocytes of male and female mice.

### Increased Adiposity of STAT5^AKO^ Mice Is Depot-Specific, but Not Correlated With Depot-Specific Changes in Adipocyte Size or BAT Activity

**Figure 2:**
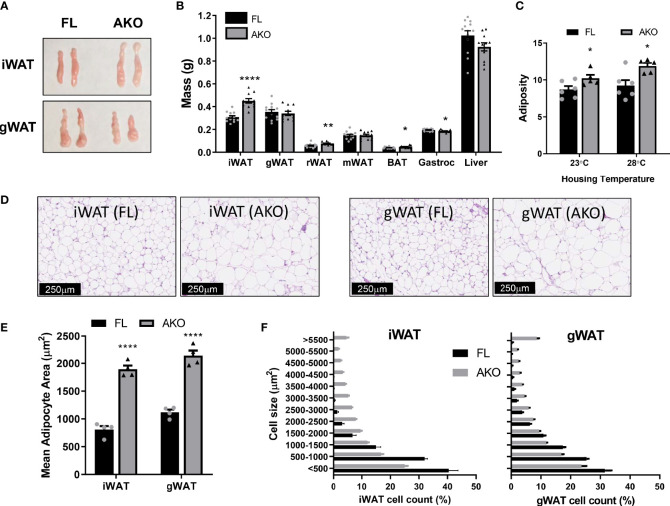
Female STAT5^AKO^ mice have increased subcutaneous adiposity when fed chow diet. Female STAT5^AKO^ (AKO) and floxed (FL) littermate control mice were weaned onto regular chow diet (13% kcal from fat). **(A)** Representative images of inguinal and gonadal white adipose tissue depots (iWAT and gWAT) from five-month-old mice. **(B)** Weights of white adipose tissue depots (iWAT, gWAT, retroperitoneal - rWAT, mesenteric - mWAT), brown adipose tissue (BAT), gastrocnemius skeletal muscle (Gastroc), and liver collected from 10-week-old mice (n = 11-13). **(C)** Mice (n = 5-6 per group) were housed at different temperatures beginning at weaning (3 weeks of age). Body composition was measured at 9 weeks of age, adiposity was calculated as fat mass divided by total body weight for each animal. **(D)** Representative images of H&E-stained inguinal and gonadal white adipose tissue depots (iWAT and gWAT) from five-month-old mice. Quantification of total mean adipocyte area **(E)** and fat cell size distribution **(F)** from H&E-stained images shown in D (n = 4 mice per genotype). Significance was determined by *t*-test and is denoted as *p < 0.05, **p < 0.01, or ****p < 0.0001 for AKO versus FL comparisons. See also **Figures S2, S3**.

As shown in **Figures 2A, B** and **S2A, B**, female and male STAT5^AKO^ mice had significantly higher iWAT weights, without discernable changes in gWAT or other fat depots, except for small statistically significant increases in retroperitoneal and brown fat. Notably, only the subcutaneous iWAT depot of STAT5^AKO^ mice was consistently larger in multiple cohorts of mice at different ages (data not shown). Histological analysis revealed hypertrophic adipocytes in both iWAT and gWAT of female (**Figures 2D–F**) and male STAT5^AKO^ mice (**Figures S2D–F**).

To assess the robustness of the adiposity phenotype in STAT5^AKO^ mice and the potential role of BAT, we also examined body composition at thermoneutrality. As shown in **Figures 2C** and **S2C**, female and male mice maintained increased adiposity following six weeks of thermoneutral housing conditions. While females demonstrated no differences in BW or lean mass at either housing temperature, male STAT5^AKO^ mice had higher fat mass at both temperatures, but higher lean mass and BW at 28°C only (**Figure S3**). Taken together, these data suggest that STAT5 signaling in BAT does not substantially contribute to development of the adiposity phenotype.

### Glucose and Lipid Metabolism in STAT5^AKO^ Mice

**Figure 3:**
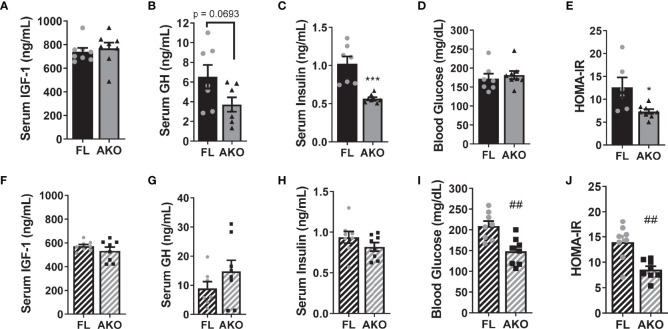
STAT5^AKO^ mice have improved homeostasis model assessment of insulin resistance (HOMA-IR). Female **(A–E)** and male **(F–J)** STAT5^AKO^ (AKO) and floxed (FL) littermate control mice were weaned onto regular chow diet (13% kcal from fat) and switched to a defined-composition LFD (10% kcal from fat) at 6 weeks of age (WOA), followed by HFD (60% kcal from fat) at 11 WOA for 4 days prior to euthanasia. Serum insulin-like growth factor 1 (IGF-1; **A, F**), growth hormone (GH; **B, G**), insulin **(C, H)**, and blood glucose **(D, I)** levels in 3-month-old mice (n = 7 – 8). **(E, J)** HOMA-IR was calculated from insulin and glucose levels, respectively in **(C, D)** or **(H, I)**. Significance was determined by *t*-test and is denoted as ***p < 0.001, for AKO versus FL comparisons in females and ^##^p < 0.01 in males.

The phenotype of animal models with altered GH signaling is often confounded by differences in IGF-1 levels. Although there was a trend towards decreased GH levels, serum IGF-1 levels were unaltered in STAT5^AKO^ females (**Figures 3A, B**). Notably, these mice had significantly lower fasting insulin levels, with no difference in glucose levels (**Figures 3C, D**). Therefore, STAT5^AKO^ females were more insulin sensitive, as indicated by HOMA-IR (**Figure 3E**), despite having higher adiposity. Like females, STAT5^AKO^ males had higher subcutaneous fat mass and improved HOMA-IR, which was not, however, driven by lower insulin levels as it was in females (**Figures 3H–J**). Assessment of HOMA-IR for the male STAT5^AKO^ mice was confounded by high blood glucose levels (**Figure 3I**) of the floxed mice since serum insulin values were not significantly different (**Figure 3H**). When glucose levels of chow-fed mice were measured, we observed no difference between male control and STAT5^AKO^ mice (data not shown). However, it is notable that while the control male mice had higher than expected blood glucose levels (**Figure 3I**), possibly due to acute high-fat-diet feeding for 4 days immediately prior to euthanasia, the STAT5^AKO^ males maintained lower glucose levels. In males, circulating IGF-1 and GH levels did not significantly differ between genotypes (**Figures 3F, G**).

**Figure 4:**
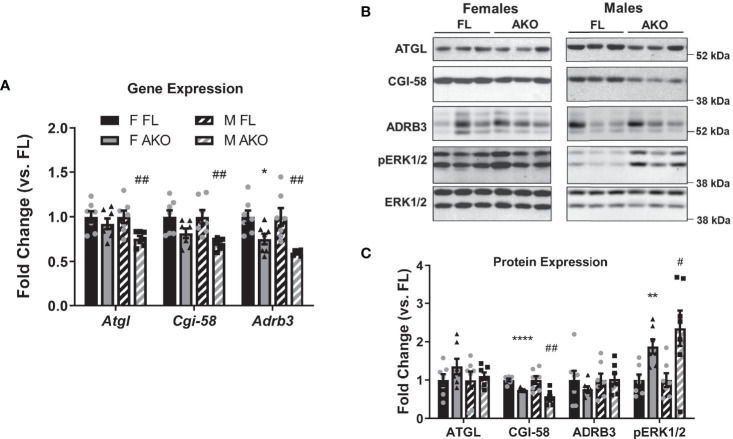
Effects of loss of adipocyte STAT5 on expression of lipolytic proteins. Chow-fed female (F) and male (M) STAT5^AKO^ (AKO) and floxed (FL) littermate control mice were euthanized at 11 weeks of age (non-fasted), and subcutaneous iWAT was collected for gene **(A)** and protein **(B, C)** expression analyses. **(A)** Gene expression of *Atgl/Pnpla2*, *Cgi-58/Abhd5*, and *Adrb3* was measured by RT-qPCR and normalized against the reference gene *Nono*. **(B)** Protein expression was examined by immunoblotting, and three representative samples per group are shown. **(C)** Band intensities were quantified and normalized against total ERK1/2 expression. Fold change was calculated as the relative gene or protein expression values divided by the mean value of the floxed control group of the same sex for each gene/protein (n = 7 per group). Significance was determined by *t*-test for FL versus AKO comparisons and is denoted as *p < 0.05, **p < 0.01, or ****p < 0.0001 for female comparisons, while significance for male comparisons is denoted as ^#^p < 0.05 or ^##^p < 0.01.

**Figure 5:**
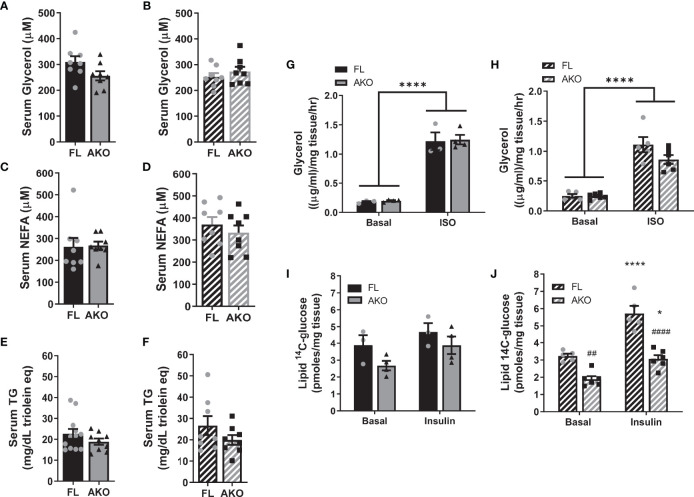
STAT5^AKO^ mice do not show any differences in adipose tissue lipid metabolism relative to floxed control mice. Female **(A, C, E, G, I)** and male **(B, D, F, H, J)** STAT5^AKO^ (AKO) and floxed (FL) littermate control mice were fed a defined-composition low-fat diet (LFD - 10% kcal from fat; **A–D**) or regular chow (13% kcal from fat; **E–J**). **(A–D)** After 1 month on LFD diet, at 3 months of age, serum glycerol and non-esterified fatty acid (NEFA) levels were measured in 4 h-fasted mice. E and F) Serum triglyceride (TG) levels were measured in 8-week-old, overnight-fasted mice. *Ex vivo* lipolysis **(G, H)** and *de novo* lipogenesis **(I, J)** assays were performed using gWAT and iWAT explants, respectively, from chow-fed mice (5 months old). **(G, H)** Glycerol release from gWAT explants (~20mg) into media following a 2 h-incubation period was measured under both basal and isoproterenol (ISO)-stimulated (10µM) conditions. I and J) Incorporation of ^14^C-glucose into total triglycerides was measured by incubating iWAT explants (~50mg) with 4µCi/ml of [^14^C]-U-glucose for 4.5 hours. The triglyceride (neutral lipid) fraction was purified and [^14^C] counts were measured by scintillation counting. **(A–F)** *t*-tests were used to test for significance between means (n = 8-12 mice per genotype). **(G–J)** Two-way ANOVA with Tukey’s *post-hoc* multiple comparison analysis was used to test for significance between genotypes and treatments (n = 3-6 mice per group). Significance is denoted as *p < 0.05 or ****p < 0.0001 for basal versus ISO or insulin comparisons and ^##^p < 0.01 or ^####^p < 0.0001 for AKO versus FL comparisons.

It is largely accepted that GH promotes fat loss *via* lipolysis, and animal models with diminished adipose tissue GH signaling have altered lipolytic responses (16–19, 36, 37). We examined gene and protein expression of several known lipolytic mediators including adipose triglyceride lipase (ATGL), CGI-58 (comparative gene identification-58), and β3 adrenergic receptor (ADRB3). As shown in **Figure 4**, male STAT5^AKO^ mice have decreased gene expression of *Atgl/Pnpla2*, *Cgi-58/Abhd5* and *Adrb3*, but diminished protein expression levels for CGI-58 only. Notably, levels of phosphoactivated extracellular signal-regulated kinase 1/2 (ERK1/2) were increased in STAT5^AKO^ mice of both sexes. While the data shown in **Figure 4** were generated from a non-fasted cohort of mice, gene and protein expression analyses of iWAT from fasted mice yielded the same results (data not shown). Lipolysis of triglycerides (TGs) results in the release of glycerol and NEFA from adipocytes into the bloodstream. Circulating levels of glycerol, NEFA, and TGs were not significantly different in STAT5^AKO^ mice of either sex versus floxed controls (**Figures 5A–F**). The serum glycerol and NEFA levels showed no genotype differences in either sex, independent of diet (LFD/HFD – **Figures 5A–D** versus chow - **Figures S4A–D**), housing temperature (**Figures S4A–D** versus **Figures S4E–H**), or fasting period (**Figures S4A–D** versus **Figures S4I–L**).

Increased adiposity of STAT5^AKO^ mice could be due to either decreased lipolysis or increased lipogenesis. To assess these processes more directly we performed *ex vivo* lipolysis and *de novo* lipogenesis assays, respectively in the gWAT and iWAT of the same cohort of mice. Basal or adrenergic-induced lipolysis altered in gWAT explants from female mice (**Figure 5G**) were not significantly different as determined by levels of glycerol released into the medium. Male STAT5^AKO^ mice had a modest, non-significant decrease in adrenergic-stimulated lipolysis (**Figure 5H**). *Ex vivo* ^14^C-glucose incorporation into iWAT TGs, a measure of lipogenesis, was not different, (**Figure 5I**) either in basal or insulin-stimulated conditions, in STAT5^AKO^ females compared to controls. In males, insulin-stimulated ^14^C-glucose incorporation into iWAT TGs was greater in controls than in STAT5^AKO^ mice (**Figure 5J**).

The convention for these assays is to normalize measurements by tissue weight as done in **Figure 5**. However, since STAT5^AKO^ mice have larger adipocytes in both gWAT and iWAT (see **Figures 2** and **S2**), normalizing by tissue weight might be confounding. Normalizing by protein or DNA content also can confound the results because adipocytes only constitute 30 – 60% of adipose tissue cellular content even though they make up approximately 80% of the volume (38–41). Since the number of adipocytes per mg of tissue would be expected to significantly differ between STAT5^AKO^ and control mice due to the significant difference in adipocyte size within adipose tissue depots, we also normalized the *ex vivo* lipolysis and lipogenesis data by adipocyte number using an estimate of adipocyte number per mg tissue, which was calculated using a formula to approximate adipocyte mass (*m_ad_*) that is described in (41). This formula assumes that each adipocyte is a perfect sphere with its volume equal to $\frac{4}{3}\pi r^{3}$ , and the same density as triolein (0.915 g/ml): $m_{ad}(in\;\mu g)=\frac{0.915}{10^{6}}\times \frac{\pi }{6}\times d^{3}=\frac{0.4791}{10^{6}}\times d^{3}$ . A statistical transformation, $3\sigma \times \bar{d}+{\bar{d}}^{3}$ replaces *d* ^3^ to account for skew in calculation due to the distribution of adipocyte sizes; $\bar{d}$ is the mean diameter calculated from the cross-sectional adipocyte areas used to create **Figures 2** and **S2**, and σ is the respective standard deviation. Adipocyte number per mg, shown in **Figures S5A, B**, was calculated by inversion of the adipocyte mass. When normalized by adipocyte number per explant, glycerol release was higher, not lower, from STAT5^AKO^ AT explants compared to floxed controls, demonstrating that AT lipolysis was not decreased in STAT5^AKO^ mice using either method of normalization (**Figures S5C, D**). Notably, STAT5^AKO^ mice of both sexes had higher levels of ^14^C-glucose lipid incorporation when the data were normalized by adipocyte number (**Figures S5E, F**) versus tissue weight, indicating that elevated *de novo* lipogenesis might confer or play a role increased adiposity in STAT5^AKO^ mice.

For the room temperature (RT)- and thermoneutrality (TN)-housed cohorts of mice that were fasted for 4h or 18h prior to tissue collection, glycerol release, normalized by tissue weight, from iWAT and gWAT explants was not significantly different between genotypes of both sexes under basal conditions or in the presence of adrenergic stimuli, isoproterenol or CL 316,243 (**Figures S6, S7**). There were a few exceptions where STAT5^AKO^ mice had significantly less glycerol release than floxed controls under adrenergic-induced lipolytic conditions (see **Figures S6A, B**, **S7E**), but these instances were not consistently observed as a function of housing temperature, fasting period, sex, or AT depot. Moreover, when the glycerol levels were normalized by adipocyte number, AT explants from STAT5^AKO^ mice did not release less glycerol than control mice under any condition (data not shown). Since insulin is an important anti-lipolytic hormone, we also assessed *ex vivo* lipolysis for each condition in the presence of insulin. As shown in **Figures S6, S7**, insulin suppressed glycerol release under adrenergic-induced, but not basal conditions, and no significant genotype differences were observed under any condition, indicating that the loss of adipocyte STAT5 does not amplify the antilipolytic effect of insulin. Taken together, these data support increased lipogenesis, but not reduced lipolysis, as a principal driver of the adiposity phenotype in STAT5^AKO^ mice.

### Sex-Specific Differences in Energy Expenditure and Adipose Tissue Gene Expression in STAT5^AKO^ Mice

**Figure 6:**
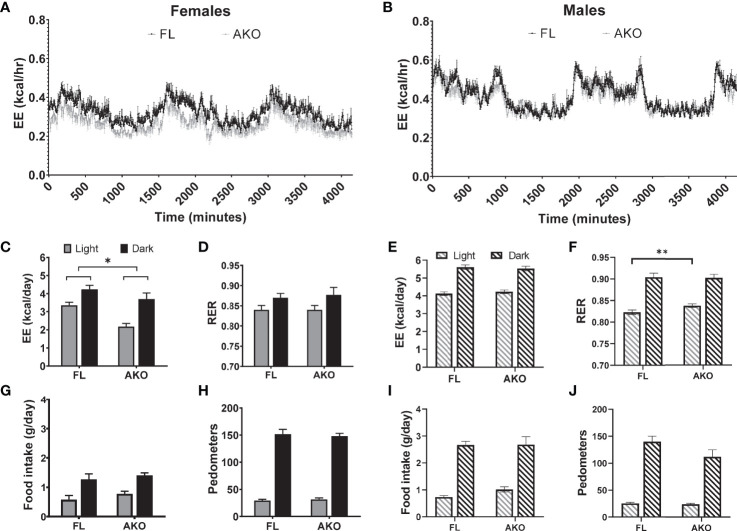
Female, but not male STAT5^AKO^ mice have reduced energy expenditure. Floxed (FL) and STAT5^AKO^ (AKO) female **(A, C, D, G, H)** and male **(B, E, F, I, J)** mice (10 weeks of age) were individually housed in metabolic cages where oxygen and carbon dioxide concentration were continuously monitored for 3 days. **(A, B, C, E)** Total energy expenditure (EE) was calculated by multiplying the daily average rate of energy expenditure by 24 and by the number of experiment days. As an estimate of substrate oxidation, **(D, F)** respiratory exchange ratio (RER, VCO2/VO2), **(G, I)** food intake and **(H, J)** total activity in walking meters (pedometers) were measured during the light and dark cycles. Significance was determined by ANCOVA with fat and fat-free masses as covariates. The * denotes p < 0.05 and **p < 0.01 (n = 8-12/group) between genotypes.

As shown in **Figures 6A, C, D**, female STAT5^AKO^ mice fed LFD had reduced daily energy expenditure when adjusted by ANCOVA for fat-free mass (see **Figure S8** for body weight and fat-free mass of mice measured in metabolic chambers) with no differences in RER between genotypes. As in all our cohorts of STAT5^AKO^ mice, there were no differences in food intake versus controls (**Figure 6G**). Mice were more active in the dark cycle, but no differences were observed between genotypes (**Figure 6H**). In a separate experiment, chow-fed male mice were also assessed in the metabolic cages. Although there were no differences in total energy expenditure between the genotypes (**Figures 6B, E**), RER was significantly increased in STAT5^AKO^ males during the light cycle (**Figure 6F**), indicating a shift toward carbohydrate utilization. Like the female mice, there were no significant differences in total daily food intake or activity between the genotypes (**Figures 6I**, **J**).

**Figure 7:**
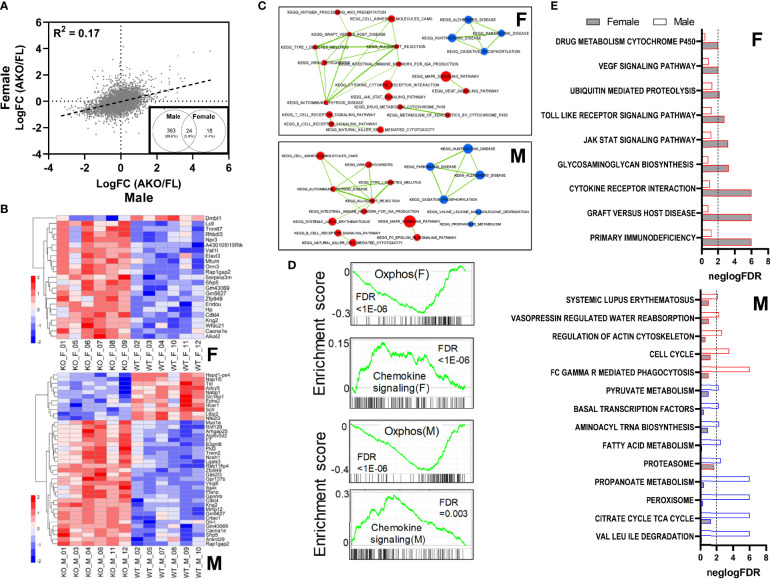
Loss of adipocyte STAT5 results in sexually dimorphic gene expression changes within subcutaneous WAT. Inguinal WAT was collected from male and emale STAT5^AKO^ and floxed (FL) control mice at 6 weeks of age, and isolated mRNA was subjected to RNA-sequencing analysis. **(A)** Scatterplot showing correlation between female and male gene expression changes resulting from adipocyte-specific loss of STAT5 (LogFC = Log fold change). The black dashed line is the trend line, and the correlation coefficient (R2) is shown. Inset: Venn diagram of 405 differentially regulated genes in male and female mice (AKO/FL) at threshold adjusted p-value equal to 0.05, expressed as numbers of genes and percentages of total regulated genes. **(B)** Heatmaps of expression levels of top differentially regulated genes in female (F) and male (M) samples. For females, genes with an adjusted p-value ≤ 0.1 and ≥ 2 or ≤ -2-fold differential gene expression, and for males, genes with adjusted p-value ≤ 0.01 and ≥ 2 or ≤ -2-fold differential gene expression are plotted. Heatmaps are row-normalized with lower gene expression shown in blue and higher gene expression in red. KO labels refer to STAT5^AKO^ mice and WT to STAT5 floxed mice. **(C)** Enrichment maps of significant pathways identified from GSEA (FDR ≤ 0.05) that share gene members. Upper and lower panels show significant pathways in females and males, respectively. Pathways upregulated in knockouts are colored in red, and downregulated pathways in blue. Thickness of lines connecting pathways is proportional to the extent of gene sharing between them. Pathways with no shared genes are not shown. **(D)** Enrichment scores of selected pathways significantly altered in both females and males, as determined *via* GSEA. The upper panels for females (F) or males (M) show enrichment plots for the ‘oxidative phosphorylation’ pathway, which was downregulated in STAT5^AKO^ mice of both sexes; bottom panel for each sex shows enrichment plot for the ‘chemokine signaling’ pathway which was upregulated in both males and females. Pathway enrichment p-values (FDR) are noted in each plot. **(E)** Pathways uniquely enriched in females (F) or males (M). For each pathway, the relative significance of enrichment (negative logarithm of the FDR) in females and males (gray and white bars, respectively) are plotted side by side, with the vertical dashed line representing an FDR of 0.01. Pathways upregulated in knockout samples are indicated by a red border and pathways downregulated in knockout samples are indicated by a blue border.

To investigate molecular mechanisms regulated by loss of STAT5 transcriptional activity in inguinal fat, we performed mRNA sequencing (RNA-Seq) on whole iWAT. The expression changes resulting from loss of adipocyte STAT5 were poorly correlated between females and males (R^2^ = 0.17) (**Figure 7A**). At an adjusted p-value ≤0.05 threshold, we identified 387 differentially regulated transcripts in males (289 upregulated and 98 downregulated), but only 42 in females (37 upregulated and 5 downregulated) (**Table S3**). Approximately 60% (24/42) of the transcripts differentially expressed in females were also regulated in males, resulting in a statistically significant overlap (Fisher exact test p < 2.2E-16) between the two lists (**Figure 7A**, inset). Unbiased principal component analysis (PCA) shows segregation of all four groups (**Figure S9**) and indicates that sex-specific differences persist in both STAT5^AKO^ mice and floxed controls. Heatmaps for the top differentially expressed genes between males and females appear in **Figure 7B**. Overall, these RNA-Seq data revealed largely sex-dependent gene expression changes in whole iWAT from STAT5^AKO^ mice, with a greater transcriptomic response observed in males but also some sex-independent changes in gene expression.

To understand the mechanistic implications of the observed differences in gene expression, we performed gene-set enrichment analysis (GSEA) (**Table S4**). Among KEGG pathways with a false discovery rate (FDR) < 0.05 we identified 8 and 21 downregulated pathways in female and male AKO samples, respectively. Of these, all 8 female-regulated pathways were also downregulated in males, and 13 were unique to males. Upregulated pathways numbered 43 and 33 in AKO female and male samples, respectively, with 21 pathways common to both sexes. The enrichment maps in **Figure 7C** show a subset of regulated pathways in female and male samples, in the form of a network based on the extent of gene sharing between pathways. The *‘oxidative phosphorylation’* pathway was downregulated in both male and female KO samples, pointing to possible disturbances in ATP-generating processes in animals lacking adipocyte STAT5. Several of the pathways upregulated in male and female KO samples were related to immune-inflammatory processes including *‘chemokine signaling’* and *‘allograft rejection’* (FDR < 1E-05 in females and 3.67E-03 in males), etc., with considerable gene sharing among the pathways (**Figure 7D**). Many lipid metabolism and fuel utilization pathways were uniquely downregulated in male KO animals, including *fatty acid metabolism, PPAR signaling, citrate cycle, propanoate metabolism, pyruvate metabolism*, etc. Interestingly, a subset of immune-inflammatory pathways was exclusively upregulated in STAT5^AKO^ females, including ‘*cytokine-cytokine receptor interaction’* (FDR <1E-05 in females and 0.10 in males), *‘JAK-STAT signaling’* (FDR 6.18E-04 in females, 0.06 in males), and *‘VEGF signaling’* (FDR 9.51E-03 in females, 0.15 in males) (**Figure 7E**). This sexual dimorphism may reflect different aspects of the inflammatory response, some common to both sexes and some unique to females.

A potential limitation of the RNA-Seq analysis is that over half of the cells in whole iWAT are not adipocytes (38, 39), and that a compensatory increase in SVF STAT5 levels was observed in our STAT5^AKO^ mice (**Figure 1E**). Therefore, although sexual dimorphism in immune-inflammatory pathways is intriguing, it is unclear whether this observation reflects changes in adipocyte gene expression or in the transcriptomes and/or proportions of co-resident immune cells.

## Discussion

STAT5 is a primary mediator of GH signaling, thus, understanding its contributions to *in vivo* adipocyte function related to metabolic health, is important. To that end, we have performed physiological studies in mice lacking both STAT5 genes in adiponectin-expressing cells. Another group has also generated this mouse model, but examined only male mice, which exhibited higher adiposity, increased adipocyte size, and evidence of improved insulin sensitivity (16). We have examined both male and female STAT5^AKO^ mice and observed increased subcutaneous adiposity in both sexes, along with indicators of improved insulin sensitivity on chow diets (**Figure 3**). Further, the following unpredicted phenotypic observations in our STAT5^AKO^ mice offer additional insight: 1) increased adiposity is not accompanied by changes in lipolysis and 2) significant sex-specific differences in energy expenditure and gene expression exist in STAT5^AKO^ mice. These observations reveal fundamental gaps in our understanding of adipocyte STAT5 and its role in metabolism.

GH regulates several metabolic pathways including lipolysis, lipogenesis, glucose uptake, and protein synthesis (42). Excess adiposity in GH-deficient patients can be improved by exogenous GH administration (43), an effect primarily attributed to GH’s lipolytic action. Male mice with adipocyte-specific deletion of GHR (36), JAK2 (17–19, 37) or STAT5 (16) have increased adiposity also attributed to decreased lipolytic rates, and impaired lipolysis in STAT5^AKO^ mice has been attributed to decreased ATGL and CGI-58 levels in subcutaneous fat (16).

In contrast to published data, our STAT5^AKO^ mice have no significant differences in circulating levels of glycerol or NEFAs, the products of lipolysis, or in *ex vivo* lipolytic activity of AT explants versus floxed controls (**Figures 5** and **S4–S7**). Therefore, decreases in lipolytic gene expression and in CGI-58 protein levels do not correlate with reduced lipolytic rates in STAT5^AKO^ AT (**Figures 4**, **5**). A variety of factors could account for the phenotypic discrepancies between the two STAT5^AKO^ lines, including genetic drift, or differences in housing conditions or gut microbiota between mouse colonies reared at different institutions. Also, our fasting conditions are limited to 4 hours or overnight, while increased lipolytic products in serum of mice lacking adipocyte GHR, JAK2, or STAT5 were observed after prolonged fasts ranging up to 48 hours (16–19, 36, 37). We specifically chose a 4h fasting period because when food intake was monitored throughout the day while the mice were in the metabolic chambers and provided food *ad libitum*, their longest period of fasting was approximately 4h. Given that the STAT5^AKO^ mice accrue more subcutaneous fat mass than the floxed control mice under *ab libitum* feeding conditions without eating more food, we believe that a 4h fasting period to examine the potential role of lipolysis in development of the adiposity phenotype is more physiologically relevant than an overnight fasting period, which would be more akin to starvation. Notably, mice lacking adipocyte-specific expression of the lipolytic mediator, ATGL, are phenotypically different from STAT5^AKO^ mice, consistent with our findings and suggesting that obesity in STAT5^AKO^ mice is not the result of reduced lipolysis.

Another consideration is that GH promotes beige fat formation *via* GHR-induced STAT activation (44). Impaired cold tolerance has been reported in STAT5^AKO^ mice (45), and the iWAT in both sexes of our STAT5^AKO^ mice appears whiter (**Figures 2A** and **S2A**), suggesting lower expression of beige adipocyte-related genes. However, we observed no alterations in expression of iWAT UCP-1 or other beiging markers (data not shown). Also, both sexes maintained higher adiposity under thermoneutral conditions (**Figures 2C** and **S2C**), suggesting that the increased fat mass of STAT5^AKO^ mice is not due to altered BAT thermogenesis.

Based on other animal models with disrupted adipose tissue GH or STAT5 signaling (16, 36, 46), we expected the increased adiposity of STAT5^AKO^ mice to be dependent on decreased lipolysis, but this was not evident in our model (**Figures 5** and **S4–S7**), despite clear evidence that adipocyte STAT5 contributes to AT deposition (**Figures 2** and **S2**). Mediators other than STAT5 have been implicated in GH-induced lipolysis, including the MEK/ERK and PLC/PKC pathways (47–49) and our STAT5^AKO^ mice exhibited increased levels of phosphoactivated ERK1/2 in iWAT (**Figure 4C**). Although we did not observe an overall decrease in AT lipolysis in STAT5^AKO^ mice, it is possible that the increased phosphorylation of ERK1/2 elevated lipolysis (50) and may have masked any lipolytic defect that might have been the direct result of loss of adipocyte STAT5. Additional experiments interrogating other molecular players within the lipolysis pathway, such as hormone sensitive lipase, perilipins, and phosphodiesterases, as well as using MEK inhibitors will be necessary to test this hypothesis.

The specificity of the subcutaneous adiposity was also intriguing and likely due to compensation within the visceral depots during development. Notably, BCL6^AKO^ mice have a very similar adiposity phenotype (51) to STAT5^AKO^ mice. Both mouse models exhibit increased subcutaneous adiposity when either BCL6 or STAT5 is knocked out of adipocytes in a congenital manner. Transcriptomics analysis revealed that both visceral (peri-gonadal) and subcutaneous WAT underwent similar and highly correlated gene expression changes with ablation of BCL6, even though only the subcutaneous depot displayed expansion in adipocyte size and fat mass. Moreover, when BCL6 was ablated only in adult mice using a doxycycline-inducible mouse model, all WAT depots (visceral and subcutaneous) exhibited increased adipocyte size and fat depot mass, supporting the hypothesis that developmental compensation drives the difference in fat mass between the depots in the BCL6^AKO^ model (51). Interestingly, BCL6 is a transcriptional target of STAT5 and these transcription factors demonstrate reciprocal occupancy within promoters to influence activation or repression of GH target genes (52). Clearly, these observations suggest some compensation among GH-induced and developmental signaling pathways and warrant further study to elucidate the specific mechanism(s) and complex crosstalk between signaling pathways and transcription factors that ultimately give rise to sex-specific gene expression and energy expenditure differences and drive the increased adiposity in our STAT5^AKO^ mice. These studies highlight STAT5^AKO^ mice as a useful tool to investigate sex-specific differences in adiposity, GH signaling, and adipose tissue depot-specific responses.

In summary, our STAT5^AKO^ mice revealed several intriguing observations. Despite the sex-independent differences in subcutaneous fat mass and adipocyte size, STAT5^AKO^ mice exhibit prominent sex-specific differences. Male and female STAT5^AKO^ mice both have higher adiposity than floxed controls, which is maintained at thermoneutrality (**Figures 2** and **S2**), indicating that BAT thermogenesis is not driving the phenotype. However, this increased fat mass is associated with decreased energy expenditure in females only (**Figure 5**). Transcriptional effects in iWAT were also sex-specific, as we observed more differentially regulated genes in males than in females. Notably, downregulation of *fatty acid metabolism, pyruvate metabolism, peroxisome, and PPAR signaling* specifically in male STAT5^AKO^ animals could indicate greater impairment of *de novo* adipogenesis than in female mice. Although STAT5 transcriptionally promotes adipogenesis in precursor cells (53–62), the use of mice with Cre expression driven by the *Adiponectin* promoter restricts STAT5 ablation to fully differentiated adipocytes. Thus, any effect of adipocyte STAT5 ablation on adipogenesis is likely indirect, and additional experiments utilizing animal models capable of tracking fat cell kinetics *in vivo* (63–66) are necessary to test this hypothesis. Taken together, our data suggest that STAT5^AKO^ mice develop increased adiposity through distinct sex-dependent mechanisms that are not consistent with defects in adipose tissue lipolysis.

## Data Availability Statement

The datasets presented in this study can be found in online repositories or **Supplementary Material**. The names of the repository/repositories and accession number(s) can be found in the article/**Supplementary Material**.

## Ethics Statement

The animal study was reviewed and approved by Institutional Animal Care and Use Committee at Pennington Biomedical Research Center.

## Author Contributions

Conceptualization, AR, CE, and JS. Methodology, AR, CE, HH, and PZ. Investigation, AR, CE, HH, PZ, and TM. Formal Analysis, AR, HH, TA, and SG. Writing – Original Draft, JS and AR. Writing – Review and Editing, all authors. Visualization, AR, HH, TA, and SG. Supervision, JS, AR, and CE. Funding Acquisition, JS and CE. All authors contributed to the article and approved the submitted version.

## Funding

This research project utilized facilities of the Genomics Core, the Cell Biology and Bioimaging Core, and the Animal Metabolism and Behavior Core at Pennington Biomedical that are supported in part by COBRE (1P30GM118430) and NORC (NIH 2P30DK072476) center grants from the National Institutes of Health. The Promethion Metabolic cage system was purchased using funds from NIH shared instrumentation grant S10OD023703. CE is supported by R03 DK122121 from NIH. This work was supported by NIH grant R01DK052968 (JS) and pilot funding to CE from a NORC center grant P30DK072476.

## Conflict of Interest

The authors declare that the research was conducted in the absence of any commercial or financial relationships that could be construed as a potential conflict of interest.

## Publisher’s Note

All claims expressed in this article are solely those of the authors and do not necessarily represent those of their affiliated organizations, or those of the publisher, the editors and the reviewers. Any product that may be evaluated in this article, or claim that may be made by its manufacturer, is not guaranteed or endorsed by the publisher.
