# Supplementary material for: Loss of Adipocyte STAT5 Confers Increased Depot-Specific Adiposity in Male and Female Mice That Is Not Associated With Altered Adipose Tissue Lipolysis
Source: Front Endocrinol (Lausanne). 2022 Apr 7;13:812802. doi: 10.3389/fendo.2022.812802 (PMC9022209; doi:10.3389/fendo.2022.812802)
Supplement: Supplementary file 1 [file DataSheet_1.pdf]

Supplementary Material

1 Supplementary Figures

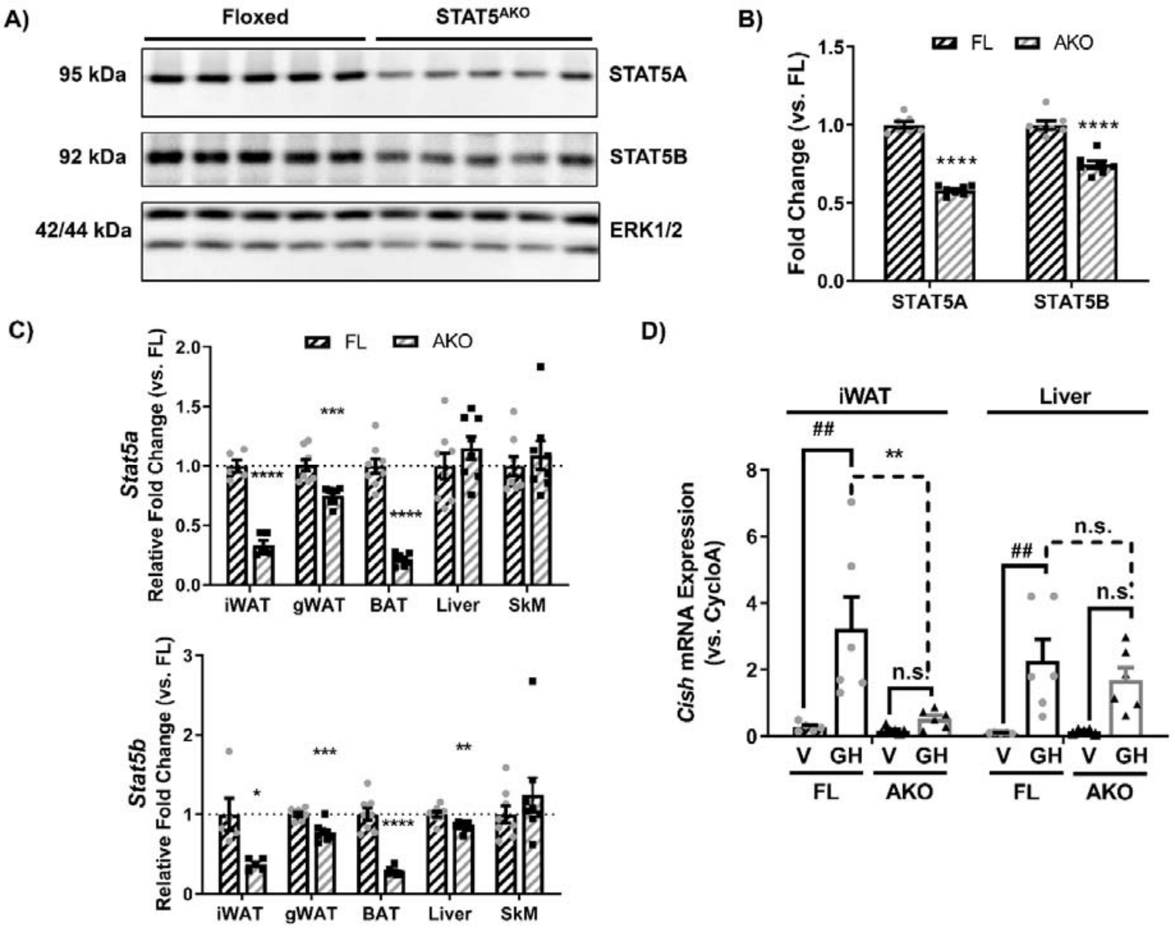

Figure S1. Expression of STAT5A and 5B is knocked down in adipose tissue and adipocytes of STAT5^{AKO} male mice. Related to Figure 1. Male STAT5^{AKO} (AKO) mice and their floxed (FL) littermate controls were euthanized at 2 – 3 months of age and tissues were immediately collected for protein or gene expression analyses. A) Immunoblot of proteins resolved from iWAT samples (n = 7 per genotype; 5 representative samples shown for each genotype). B) Quantification of band intensities from A (n = 7 per group). Band intensities were normalized to the loading control, ERK1/2, and are presented as fold change relative to FL mice. C) *Stat5a (top)* and *Stat5b (bottom)* gene expression measured by RT-qPCR for the indicated tissues (n = 5 – 8 mice per group). D) Mice were injected with 1.5mg/kg mGH or vehicle (V; 0.9% Saline-NaOH) for 30 minutes prior to euthanasia and tissue collection. *Cish* gene expression, measured by RT-qPCR, is shown (n = 5 – 6 mice per group). Significance was determined by *t*-test for AKO versus FL comparisons and is denoted as * $p < 0.05$, ** $p < 0.01$, *** $p < 0.001$, **** $p < 0.0001$, n.s., not significant. For D, a 2-way ANOVA was used to assess treatment/genotype and tissue variables with Tukey's post-hoc multiple comparisons test to compare all treatment/genotype groups for each tissue; ## denotes $p < 0.01$ for GH versus V comparisons.

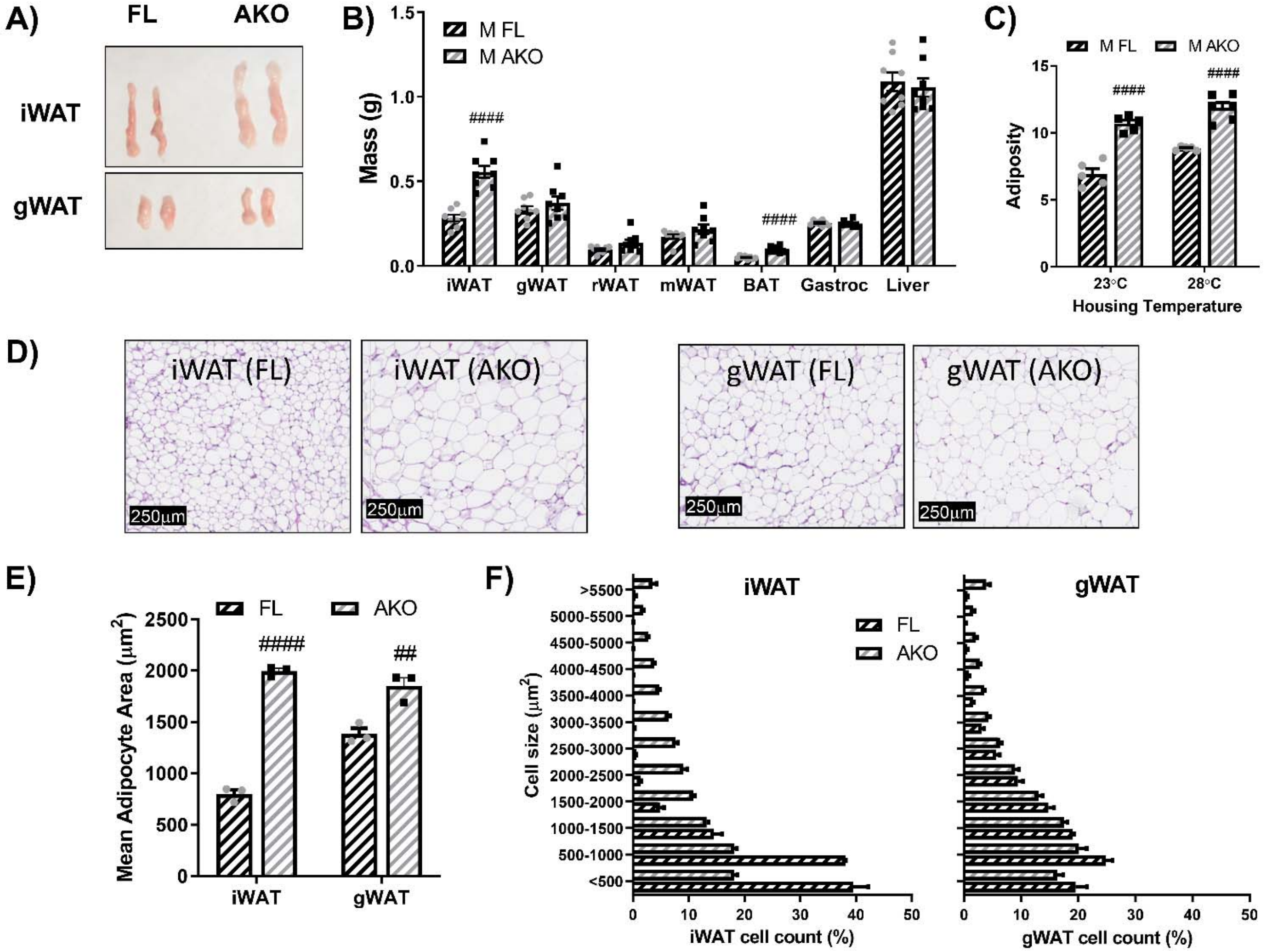

Figure S2. Male STAT5^{AKO} mice have increased subcutaneous adiposity when fed chow diet. Related to Figure 2. Male STAT5^{AKO} (AKO) and floxed (FL) littermate control mice were weaned onto regular chow diet (13% kcal from fat). A) Representative images of inguinal and gonadal white adipose tissue depots (iWAT and gWAT) from five-month-old mice. B) Weights of white adipose tissue depots (iWAT, gWAT, retroperitoneal - rWAT, mesenteric - mWAT), brown adipose tissue (BAT), gastrocnemius skeletal muscle (Gastroc), and liver from 10-week-old mice (n = 11-13). C) Mice (n = 5-6 per group) were housed at different temperatures beginning at weaning (3 weeks of age). Body composition was measured at 9 weeks of age, adiposity was calculated as fat mass divided by total body weight for each animal. D) Representative images of H&E-stained inguinal and gonadal white adipose tissue depots (iWAT and gWAT) from five-month-old mice. Quantification of total mean adipocyte area (E) and fat cell size distribution (F) from H&E-stained images shown in D (n = 3 mice per genotype). Significance was determined by *t*-test and is denoted as ## $p < 0.01$ or #### $p < 0.0001$ for AKO versus FL comparisons.

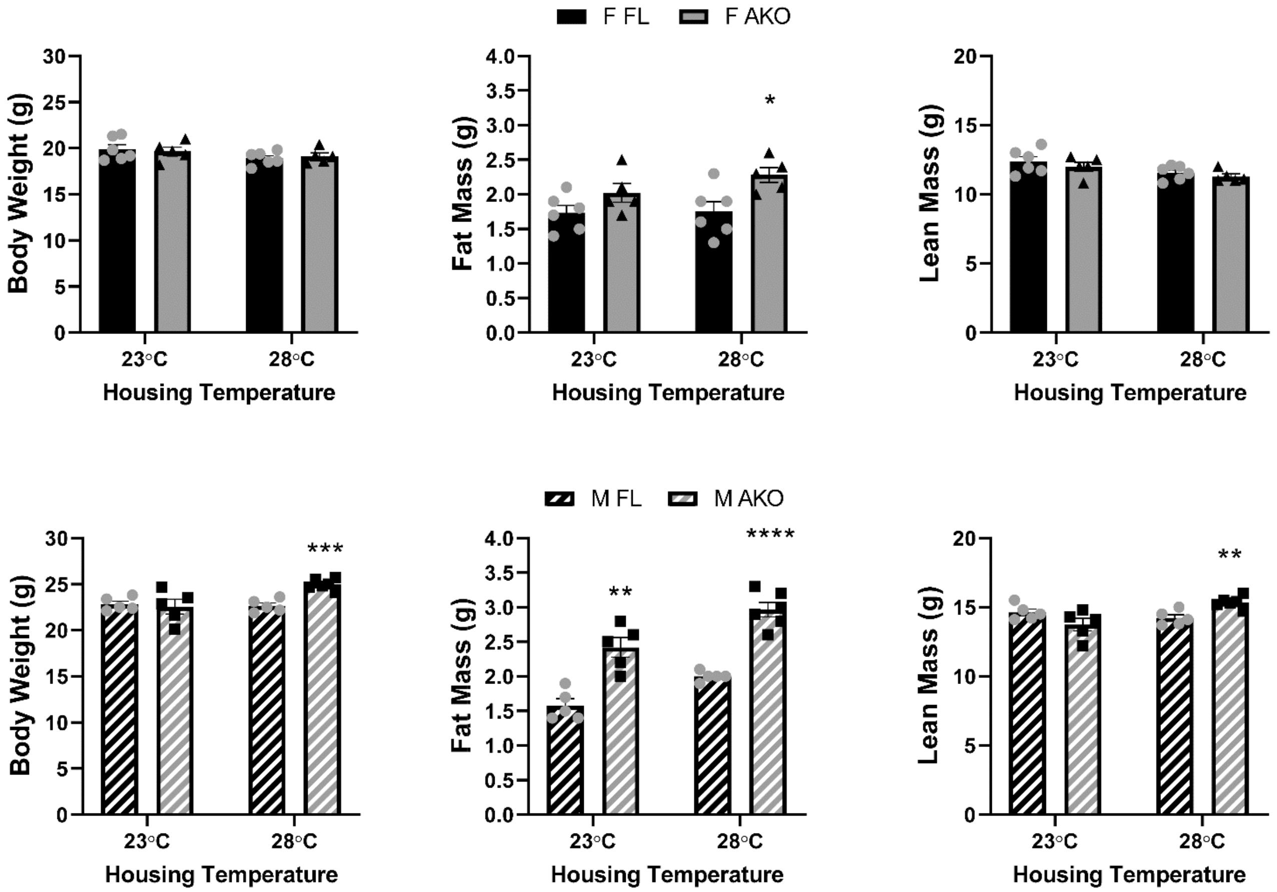

Figure S3. Body composition data for mice at ambient and thermoneutral housing temperatures. Related to Figures 2 and S2. Female (top) and male (bottom) mice (n = 5 – 6 per group) were housed at the indicated temperatures beginning at weaning (3 weeks of age) and fed chow diet (13% kcal from fat). Body weight and body composition (fat mass and lean mass measured by NMR) were assessed at 9 weeks of age. Significance was determined using multiple *t*-tests with the Holm-Sidak method, and the adjusted p-value is denoted as * $p < 0.05$, ** $p < 0.01$, *** $p < 0.001$, or **** $p < 0.0001$ for AKO versus FL comparisons.

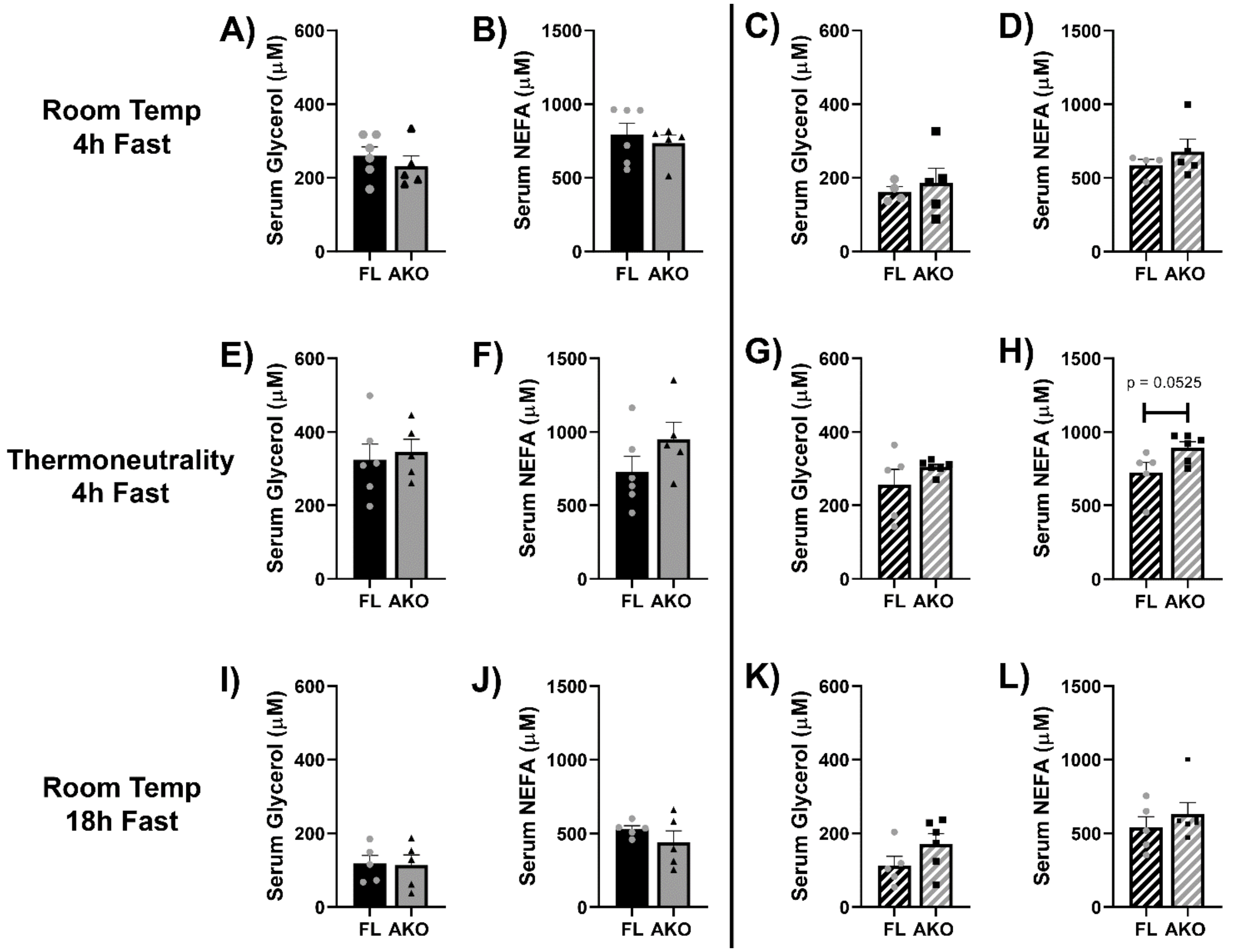

Figure S4. Serum glycerol and non-esterified fatty acids (NEFA) measurements from mice that were housed at room temperature (23°C) or thermoneutrality (28°C) and fasted for 4h or 18h. Related to Figure 5. Chow-fed female (A, B, E, F, I, and J) and male (C, D, G, H, K, and L) $STAT5^{AKO}$ (AKO) and floxed (FL) littermate control mice were housed at 23°C (room temp) or 28°C (thermoneutrality) upon weaning at 3 weeks of age (WOA). At 9 WOA, blood was collected via cardiac puncture following a 4h or 18h fast, and serum glycerol and NEFA levels were measured. Student's *t*-tests were used to test for significance between means (n = 4 - 6 mice per genotype). None of the comparisons reached significance ($p < 0.05$).

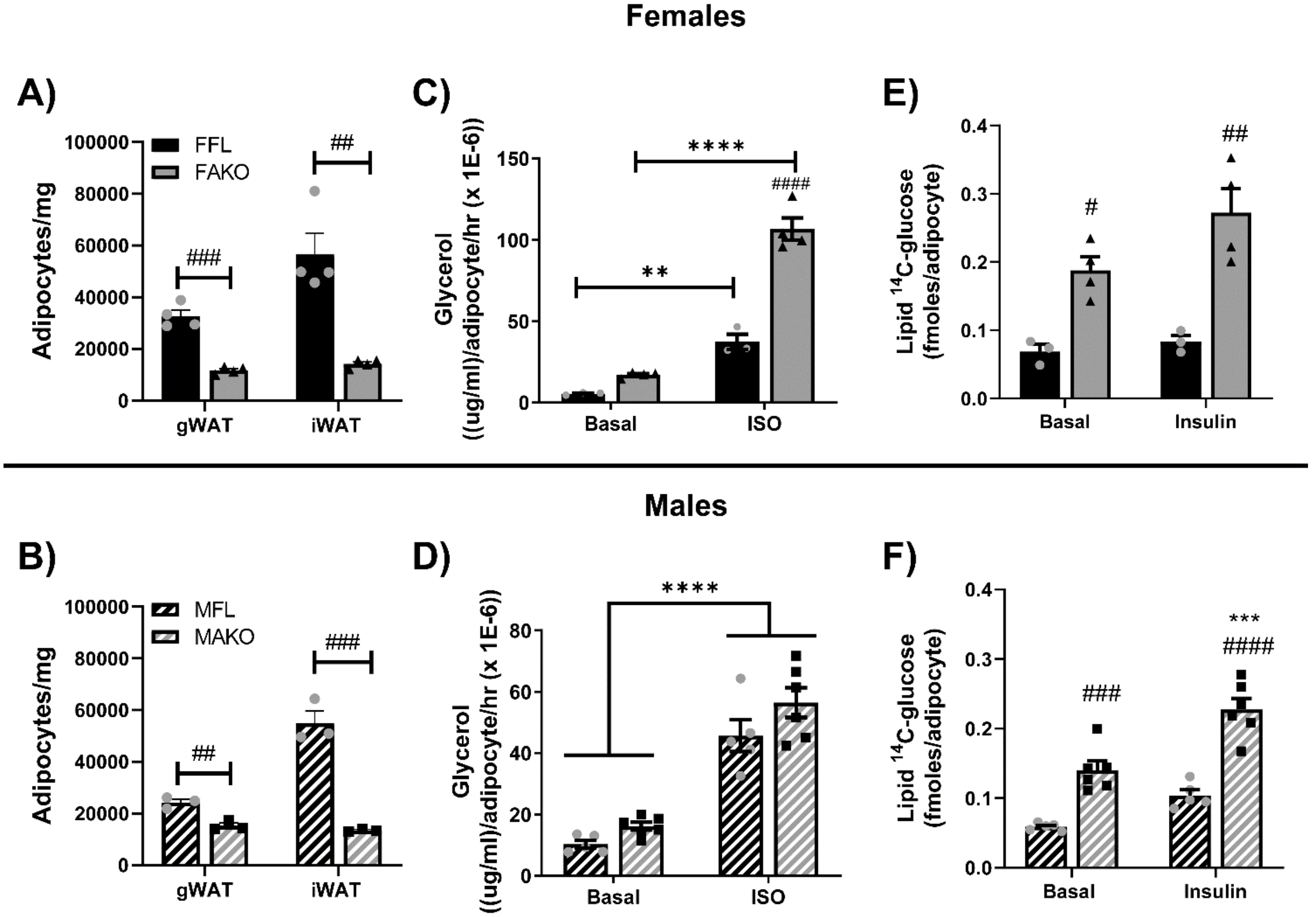

Figure S5. Normalization of *ex vivo* assay data by adipocyte number/explant. Related to Figure 5. *Ex vivo* lipolysis (C and D) and *de novo* lipogenesis (E and F) assays were performed using gWAT and iWAT explants, respectively, from chow-fed 5-month-old female (top) and male (bottom) $STAT5^{AKO}$ (AKO) and floxed (FL) littermate mice following a 4h fast. A and B) Using approximate adipocytes diameters, which were calculated from the cross-sectional area data used to generate Figures 2 and S2 D-FG, an estimated number of adipocytes per mg tissue was calculated for each AT depot as described in Section 3.3 of the Results. C and D) Glycerol release from gWAT explants (~20mg) into media following a 2 h-incubation period was measured under both basal and isoproterenol (ISO)-stimulated (10µM) conditions. E and F) Incorporation of ^{14}C-glucose into total triglycerides was measured by incubating iWAT explants (~50mg) with 4µCi/ml of [^{14}C]-U-glucose for 4.5 hours. The triglyceride (neutral lipid) fraction was purified and [^{14}C] counts were measured by scintillation counting. All *ex vivo* assay data were normalized by an estimate of adipocyte number per explant. A and B) *t*-tests were used to test for significance between means (n = 3 - 4 mice per genotype). C and D) Two-way ANOVA with Tukey's post-hoc multiple comparison analysis was used to test for significance between genotypes and treatments (n = 3 - 6 mice per group). Significance is denoted as ** $p < 0.01$, *** $p < 0.001$, or **** $p < 0.0001$ for basal versus ISO or insulin comparisons and # $p < 0.05$, ## $p < 0.01$, ### $p < 0.001$, or #### $p < 0.0001$ for AKO versus FL comparisons.

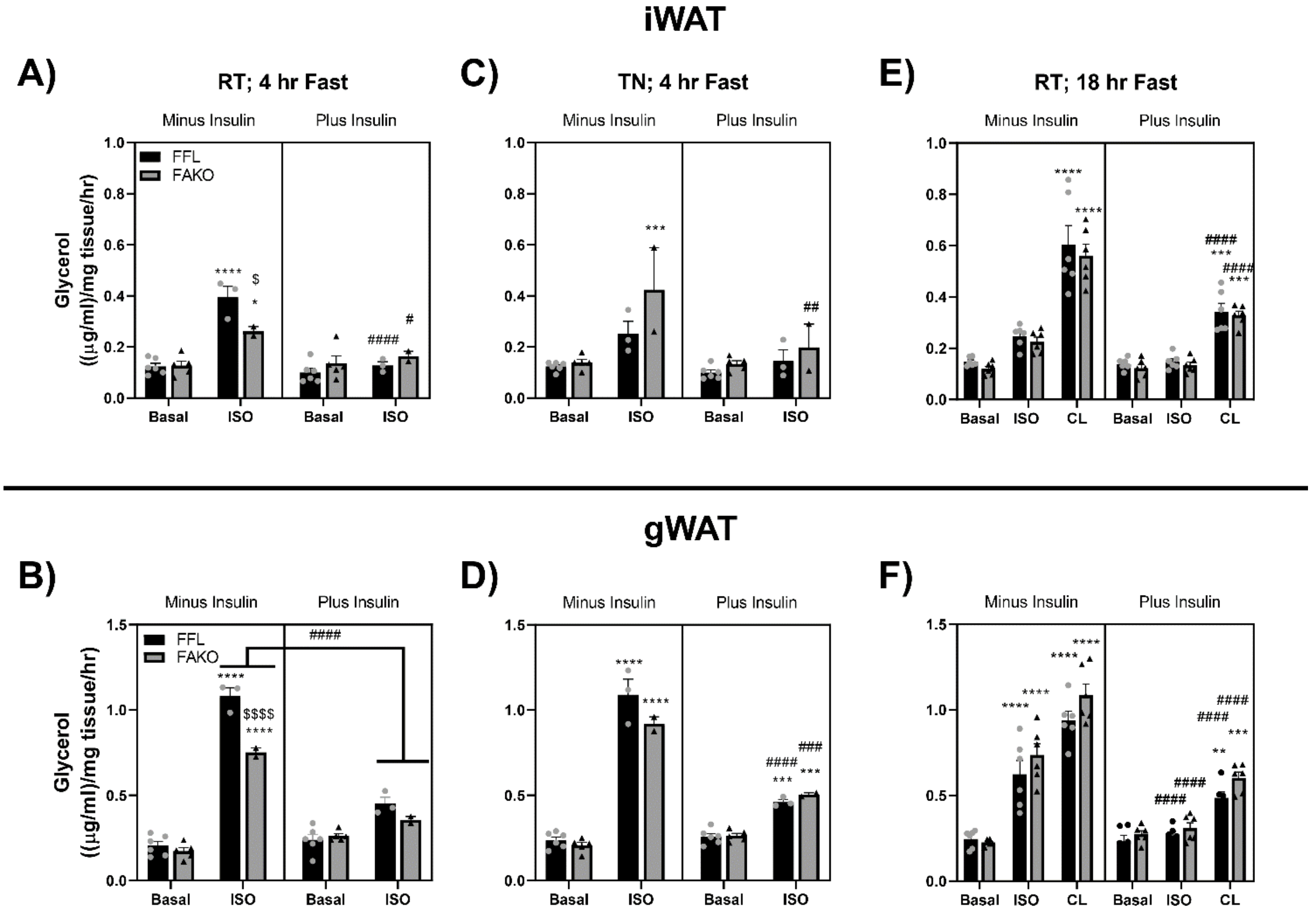

Figure S6. Glycerol release during *ex vivo* lipolysis assays performed on AT explants from female mice. Related to Figure 5. Chow-fed male STAT5^{AKO} (AKO) and floxed (FL) littermate control mice were housed at 23°C (room temp; RT) or 28°C (thermoneutrality; TN) upon weaning at 3 weeks of age (WOA). At 9 WOA, mice were euthanized following a 4h or 18h fast, and *ex vivo* lipolysis assays were performed on iWAT and gWAT explants. Conditioned media were collected following incubation of the AT explants (~20mg) with water vehicle (basal), isoproterenol (ISO; 1µM for A – D and 200nM for E - F), or CL 316,243 (CL; 200nM for E and F) for 2h. The explants were rinsed in phosphate buffer saline and the transferred to fresh medium containing the vehicle, ISO, or CL treatments as before plus 10nM insulin, and then conditioned media were collected following a 1h incubation period. All glycerol levels were normalized by tissue weight and incubation time. A three-way, repeated measures ANOVA with Bonferroni's post-hoc multiple comparison analysis was used to test for significance between genotypes and treatments with insulin treatment as the repeated measure (n = 2 - 6 mice per group). Significance is denoted as * $p < 0.05$, ** $p < 0.01$, *** $p < 0.001$, or **** $p < 0.0001$ for basal versus ISO or CL comparisons within the same genotype and insulin condition; # $p < 0.05$, ## $p < 0.01$, ### $p < 0.001$, or #### $p < 0.0001$ for minus versus plus insulin comparison of same genotype and treatment condition; and \$ $p < 0.05$ or \$\$\$\$ $p < 0.0001$ for AKO versus FL comparisons.

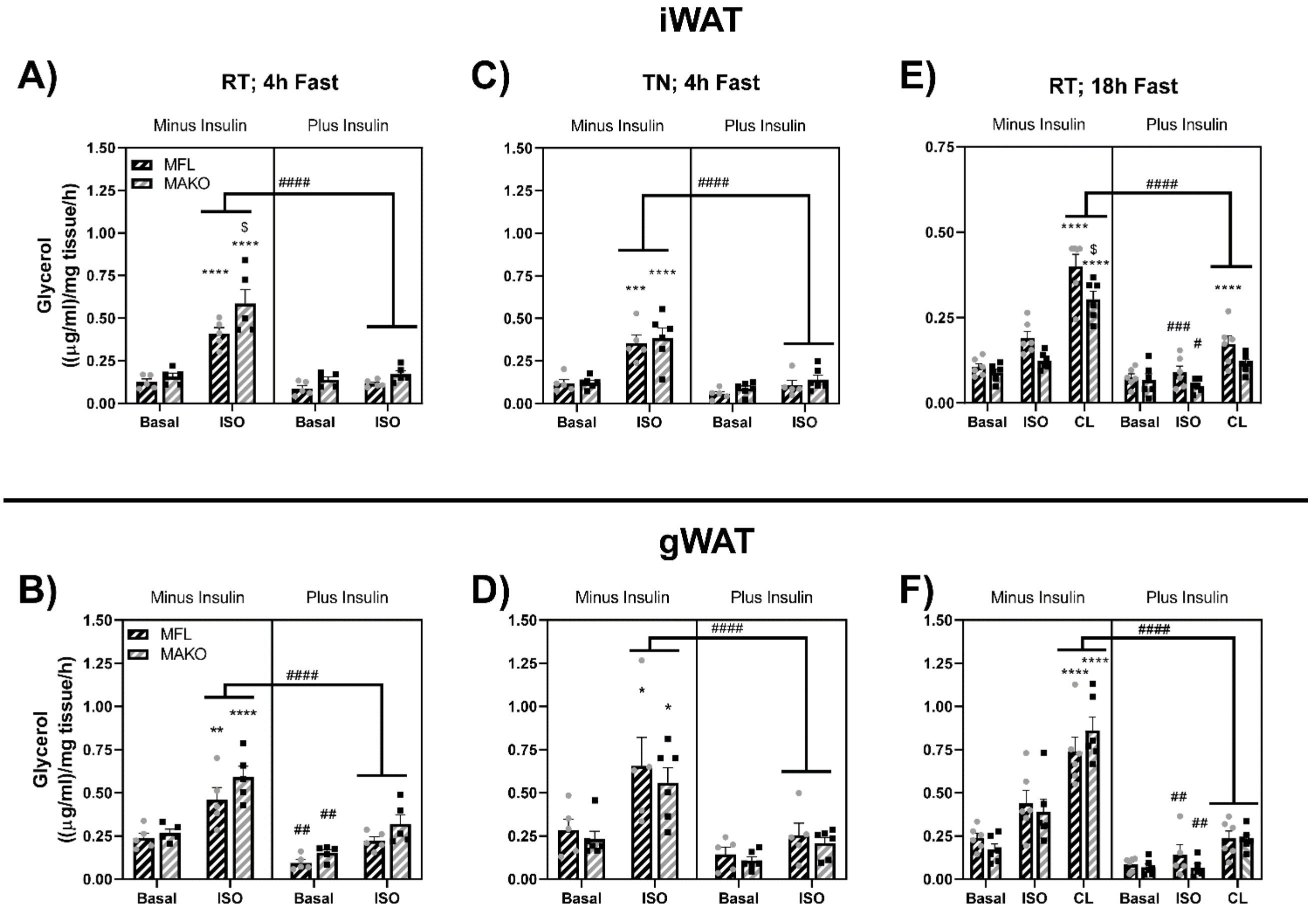

Figure S7. Glycerol release during *ex vivo* lipolysis assays performed on AT explants from male mice. Related to Figure 5. Chow-fed male STAT5^{AKO} (AKO) and floxed (FL) littermate control mice were housed at 23°C (room temp; RT) or 28°C (thermoneutrality; TN) upon weaning at 3 weeks of age (WOA). At 9 WOA, mice were euthanized following a 4h or 18h fast, and *ex vivo* lipolysis assays were performed on iWAT and gWAT explants. Conditioned media were collected following incubation of the AT explants (~20mg) with water vehicle (basal), isoproterenol (ISO; 1µM for A – D and 200nM for E - F), or CL 316,243 (CL; 200nM for E and F) for 2h. The explants were rinsed in phosphate buffer saline and the transferred to fresh medium containing the vehicle, ISO, or CL treatments as before plus 10nM insulin, and then conditioned media were collected following a 1h incubation period. All glycerol levels were normalized by tissue weight and incubation time. A three-way, repeated measures ANOVA (A – E) or a mixed-effect model (F) with Bonferroni's post-hoc multiple comparison analysis was used to test for significance between genotypes and treatments with insulin treatment as the repeated measure (n = 5 - 6 mice per group). Significance is denoted as * $p < 0.05$, ** $p < 0.01$, *** $p < 0.001$, or **** $p < 0.0001$ for basal versus ISO or CL comparisons within the same genotype and insulin condition; # $p < 0.05$, ## $p < 0.01$, ### $p < 0.001$, or #### $p < 0.0001$ for minus versus plus insulin comparison of same genotype and treatment condition; and $ $p < 0.05$ for AKO versus FL comparisons.

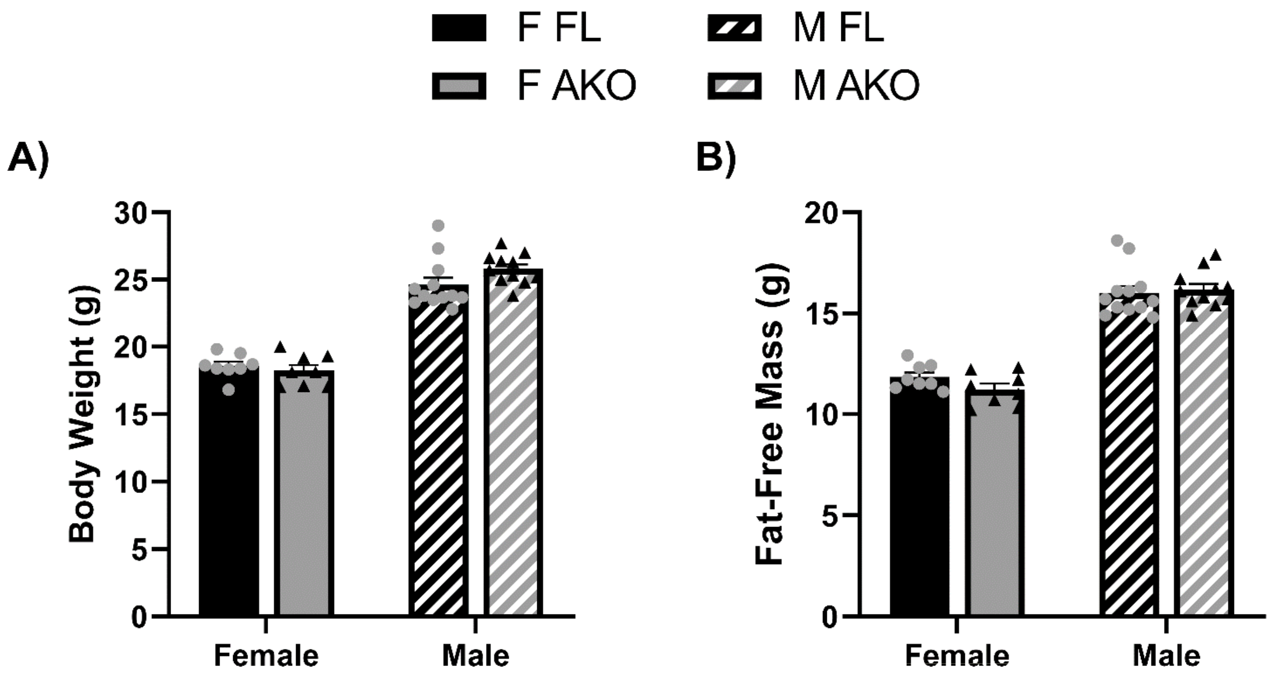

Figure S8. Body weight and fat-free mass of female (F) and male (M) floxed control (FL) and STAT5^{AKO} (AKO) mice measured in metabolic chambers. Related to Figure 6. Body weight (A) and fat-free mass (B) were measured within 24 hours prior to placing the mice in the metabolic chambers. Significance was determined using multiple *t*-tests with the Holm-Sidak method, and none of the FL versus AKO comparisons were significantly different.

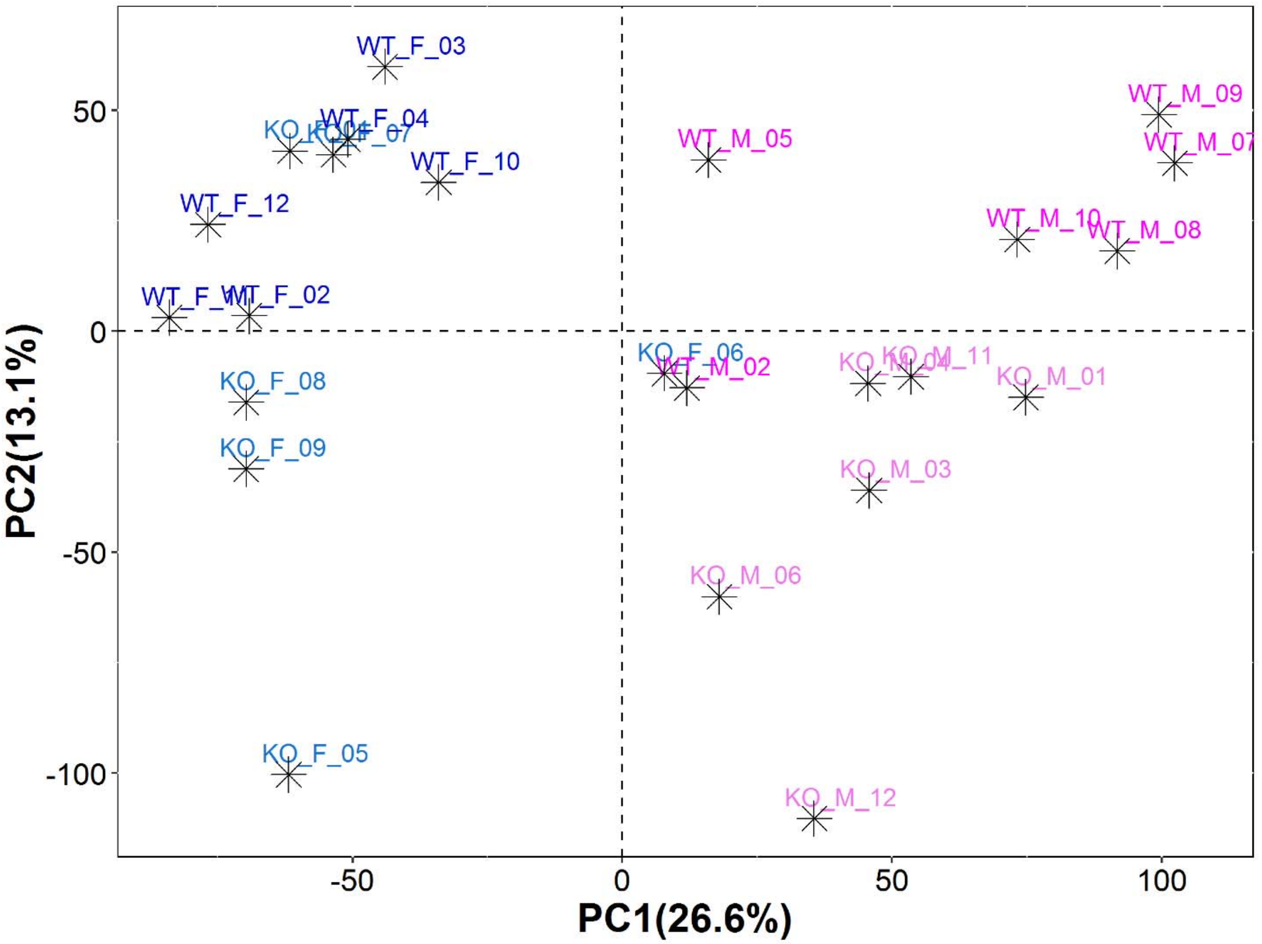

Figure S9. Principal Components Analysis (PCA) scatterplot of RNA-sequencing gene expression data from male (M) and female (F) floxed (WT) and STAT5 adipocyte knockout (KO) mice. Related to Figure 7. PCA was performed on voom-adjusted log2 cpm data, after filtering out low expressed genes in limma. Each point represents data from an individual mouse. Floxed females - dark blue, KO females - light blue, floxed males – dark pink, and KO males – light pink.

2 Supplementary Tables

Table S1. Primer sequences for qPCR.

Gene	Primer 1 (5' – 3')	Primer 2 (5' – 3')
Cgi-58/Abhd5	CCCACATCTACATCACACCTT	GAGAGAACATCAGCGTCCATA
Adrb3	CCACCGCTCAACAGGTTT	CCAGAAGTCCTGCAAAAACG
Cish	GCTCCTTTCTCCTTCCATCC	CCGCCCAATTTGCTCCA
Atgl/Pnpla2	GAGCTCATCCAGGCCAAT	CTCATAAAGTGGCAAGTTGTCTG
Stat5a	**	**
Stat5b	GTTCAACATCAGCAGCAACC	TCAATACTTCCATCACGCCATC
Nono	CATCATCAGCATCACCACCA	TCTTCAGGTCAATAGTCAAGCC
Cyclophilin a (Ppia)	TCTTCAGGTCAATAGTCAAGCC	TGCAAACAGCTCGAAGGAGACGC

** Qiagen RT^2 qPCR Primer Assay for Mouse Stat5a – Cat No. PPM04026C-200

Table S2. Key resources table.

REAGENT or RESOURCE	SOURCE	IDENTIFIER
Antibodies		
STAT5A	Santa Cruz Biotechnology<br>Abcam	Cat#: sc-1081 (discontinued); RRID: AB_632448<br>Cat#: ab32043; RRID: AB_778107
STAT5B	R&D	Cat#: AF1584; RRID: AB_2197076
Adiponectin (ADPN)	Thermo Fisher Scientific	Cat#: PA1-054; RRID: AB_325789
ATGL (PNPLA2)	Cell Signaling Technology	Cat#: 2439: RRID: AB_2167953
CGI-58 (ABHD5)	Santa Cruz Biotechnology	Cat#: sc-376931; RRID: AB_2868519
ADRB3	Santa Cruz Biotechnology	Cat#: sc-515763; RRID: AB_2868520
pERK1/2 (pTEpY)	Promega	Cat#: V8031; RRID: AB_430866
ERK1/2	Santa Cruz Biotechnology<br>Cell Signaling Technology	Cat#: sc-93 (discontinued); RRID: 631453<br>Cat#: 4695; RRID: AB_390779
Anti-rabbit HRP-conjugated	Jackson ImmunoResearch	Cat#: 111-035-003
Anti-mouse HRP-conjugated	Jackson ImmunoResearch	Cat#: 115-035-071
Chemicals, Peptide, and Recombinant Proteins		
Acrylamide, ProtoGel (30%)	National Diagnostics	EC-890
Collagenase, Type I	Worthington Biochemical	LS004196
(-)-Isoproterenol (+)-bitartrate	Sigma-Aldrich	I2760
Murine growth hormone	National Hormone and Peptide program	AFP904
Insulin from bovine pancreas (for lipogenesis assays)	Sigma-Aldrich	I5500
Formalin	Thermo Fisher Scientific	5725
TRIzol Reagent	Thermo Fisher Scientific	15596018
Critical Commercial Assays/Kits		
SuperSignal West Pico PLUS Chemiluminescent Substrate	Thermo Fisher Scientific	34578
Glycerol Assay Kit<br>Glycerol Standard	Sigma-Aldrich	KG0100<br>G7793
NEFA Assay Kit	BioVision<br>Wako diagnostics	K612-100<br>99934691, 99534791, 99134891, 99335191, 27676491
Triglyceride Assay Kit	Sigma-Aldrich	TR0100
Mouse Insulin ELISA	Crystal Chem	90080
Mouse IGF-1 ELISA	Crystal Chem	80574
Mouse GH ELISA	Millipore Sigma	EZRMGH-45K
SYBR® Premix Ex Taq (Tli RNase H Plus), Rox Plus	Takara Bio USA	RR42LR
Quant-Seq 3' mRNA-Seq Library Prep Kit FWD for Illumina	Lexogen	015.2X96
Deposited Data		
Raw and analyzed RNA-seq data	This paper	GEO: GSE113939
Experimental Models: Organisms/Strains		
B6.FVB-Tg(Adiopq-cre)1Evdr/J	The Jackson Laboratory	028020
$STAT5^{fl/fl}$	(Cui et al., 2004)	N/A
$STAT5^{AKO}$	This paper	N/A

Oligonucleotides		
Primers for qPCR	Table S1	N/A
Software and Algorithms		
Graph Pad Prism 6 & 8.4	GraphPad Software	N/A
JMP v. 14	SAS Institute Inc.	
Image Studio Lite Ver 5.2	LI-COR Biosciences	N/A
NanoZoomer Digital Pathology; NDP.view 2.7.52	Hamamatsu	N/A
QuantSeq pipeline V1.8.8 on Bluebee platform	https://www.lexogen.com/store/quantseq-data-analysis-bluebee-platform/	N/A
Other		
Chow Diet	LabDiet®	5001 (standard) and 5015 (breeder)
Low Fat Diet (LFD), no sucrose	Research Diets	D12450K
High Fat Diet (HFD)	Research Diets	D12492
IACUC Protocol Numbers	n/a	P863, P977, P985
Glucometer	Bayer	Breeze 2
Sarstedt Capillary blood collection tube	Fisher Scientific	NC9059691
Microplate spectrophotometer	Molecular Devices	VersaMax
NMR Machine	Bruker	Minispec LF110
7900HT qPCR Machine	Applied Biosystems	N/A
Metabolic Chambers	Sable Systems International	Promethion
NextSeq 500 Sequencer	Illumina	N/A

Table S3. Differentially expressed genes in male and female STAT5^{AKO} mice from RNA sequencing analysis.

Table S3.xlsx

Table S4. Differentially regulated KEGG pathways identified by gene-set enrichment analysis (GSEA).

Table S4.xlsx
